# Nonlinear optical property measurements of rhenium diselenide used for ultrafast fiber laser mode-locking at 1.9 μm

**DOI:** 10.1038/s41598-021-88735-1

**Published:** 2021-04-29

**Authors:** Jinho Lee, Suhyoung Kwon, Taeyoon Kim, Junha Jung, Luming Zhao, Ju Han Lee

**Affiliations:** 1 School of Electrical and Computer Engineering, University of Seoul, Seoul, 02504 South Korea; 2 School of Optical and Electronic Information and Wuhan National Laboratory for Optoelectronics, Huazhong University of Science and Technology, Wuhan, 430074 China; 3 Institute of Information Technology, University of Seoul, Seoul, 02504 South Korea

**Keywords:** Optics and photonics, Physics

## Abstract

An experimental investigation into the nonlinear optical properties of rhenium diselenide (ReSe_2_) was conducted at a wavelength of 1.9 μm using the open-aperture and closed-aperture Z-scan techniques for the nonlinear optical coefficient (β) and nonlinear refractive index (*n*_2_) of ReSe_2_, respectively. β and *n*_*2*_ measured at 1.9 μm were ~ − 11.3 × 10^3^ cm/GW and ~ − 6.2 × 10^–2^ cm^2^/GW, respectively, which to the best of our knowledge, are the first reported measurements for ReSe_2_ in the 1.9-μm spectral region. The electronic band structures of both ReSe_2_ and its defective structures were also calculated via the Perdew–Becke–Erzenhof functional to better understand their absorption properties. A saturable absorber (SA) was subsequently fabricated to demonstrate the usefulness of ReSe_2_ for implementing a practical nonlinear optical device at 1.9 μm. The 1.9-μm SA exhibited a modulation depth of ~ 8% and saturation intensity of ~ 11.4 MW/cm^2^. The successful use of the ReSe_2_-based SA for mode-locking of a thulium–holmium (Tm–Ho) co-doped fiber ring cavity was achieved with output pulses of ~ 840 fs at 1927 nm. We believe that the mode-locking was achieved through a hybrid mechanism of saturable absorption and nonlinear polarization rotation.

## Introduction

Nonlinear optical materials have been an essential part of realizing various nonlinear functions in optical and photonic devices, such as frequency conversion^1,2^, parametric processes^3^, optical switching^4^, and saturable absorption^5^. The aforementioned nonlinear optical functions require different nonlinear optical effects of the material; for example, the χ^(2)^ effect is used for the implementation of optical wavelength converters^6^, χ^(3)^ is for optical switches^7^, and nonlinear absorption is for saturable absorbers (SAs)^8^.

SAs can be broadly categorized into fast and slow devices. It is well-known that fast SAs are suitable for generating ultrashort pulses owing to their immediate response time^9^. Optical fiber-based nonlinear transmission devices comprising a nonlinear optical loop mirror (NOLM), a nonlinear amplifying loop mirror, or nonlinear polarization rotation (NPR) are commonly known as fast SAs^10–12^. However, mode-locking with the aforementioned optical fiber-based nonlinear devices has a self-starting difficulty. On the other hand, one of the latest developments in the field of materials science, nonlinear saturable absorption materials, are known to be relatively slow SAs compared to optical fiber-based nonlinear transmission devices. The saturable absorption phenomenon usually occurs because of Pauli’s blocking principle within semiconductors^13^. It is well-known that nonlinear material-based SAs are suitable for self-starting mode-locking operations. Until now, many nonlinear optical materials can be used as relatively slow SAs, such as carbon nanotubes^5,14,15^, graphene^8,16,17^, topological insulators (TIs)^18,19^, transition metal dichalcogenides (TMDCs)^20–25^, lead sulfide (PbS)^26^, and MXenes^27–29^.

Hybrid mode-locking techniques have been widely investigated to solve the difficulty with self-starting for conventional mode-locking in optical fiber-based nonlinear transmission devices. They can induce self-starting mode-locking with ultrafast and stable pulses by combining an optical fiber-based nonlinear transmission device and a nonlinear material-based SA: the former can induce ultrashort pulse duration while the latter can easily initiate self-starting for the mode-locking process to enhance the overall mode-locking stability.

Among the saturable absorption materials, TMDCs have received much attention. TMDCs have the chemical formula MX_2_ (M: transition metal, X: chalcogen). Zhang et al.^20^ first investigated the saturable absorption property of molybdenum disulfide (MoS_2_), after which this property has been extensively investigated in various other TMDCs^20–25^.

Compared to conventional group VI TMDCs (molybdenum- or tungsten-based ones), rhenium-based ones (group VII TMDCs) are known to possess quite different optical and electronic characteristics. Very recently, several rhenium-based TDMCs, such as rhenium disulfide (ReS_2_) and ReSe_2_, for use as nonlinear SAs have been reported^30–34^. These SAs have been implemented in Q-switching^31,32^ or mode-locking^33,34^, and their nonlinear absorption coefficients have been obtained using Z-scan techniques conducted at wavelengths of 515, 1030, or 1560 nm^33,34^.

In this work, we measured the nonlinear optical properties of ReSe_2_ at a wavelength of 1.9 μm. First, the nonlinear absorption coefficient (β) of ReSe_2_ was measured using the open-aperture (OA) Z-scan technique (~ − 11.3 $\times$ 10^3^ cm/GW). Next, the nonlinear refractive index (*n*_*2*_) of ReSe_2_ was measured using the closed-aperture (CA) Z-scan technique (~ − 6.2 $\times$ 10^–2^ cm^2^/GW). To the best of our knowledge, these are the first measurements of β and *n*_2_ of ReSe_2_ in the 1.9-μm spectral region. Density functional theory (DFT) calculations were conducted to determine the electronic band structures of ReSe_2_ for different ratios of Re and Se atoms. Finally, a composite of ReSe_2_ and polyvinyl alcohol (PVA) was fabricated to assess the practicability of using ReSe_2_ as an SA at 1.9 μm. Ultrafast ~ 840 fs pulses could readily be obtained after incorporating the prepared SA into a 1.9 μm fiber ring cavity as a mode-locker.

## Results

### Material characterization and bandgap calculation of ReSe_2_ particles

**Figure 1:**
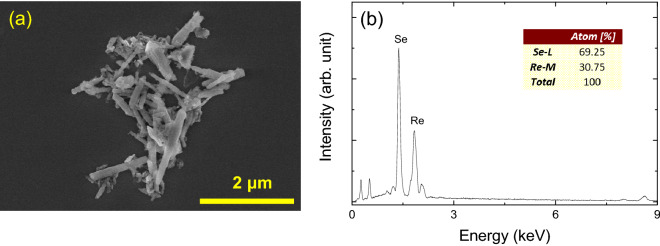
(**a**) An SEM image and (**b**) an EDS spectrum of ReSe_2_ particles.

ReSe_2_ bulk flakes were first suspended in 10 mL distilled water, after which the solution was sonicated for 16 h to obtain nano-sized particles. Scanning electron microscopy (SEM) was conducted on the sonicated particles, and the SEM image in Fig. 1a clearly illustrates that the particle size varied from hundreds of nanometers to a few micrometers, indicating that the ReSe_2_ particles were still in the bulk state.

**Figure 2:**
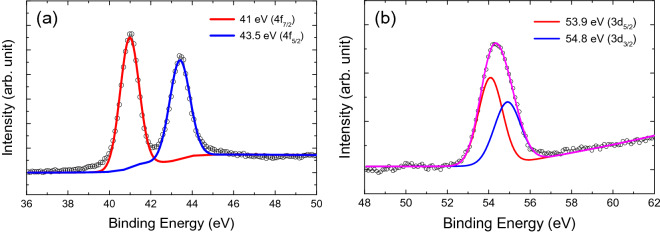
XPS core-level spectra: (**a**) Re 4f and (**b**) Se 3d.

Characterization of the sonicated ReSe_2_ particles was conducted via energy dispersive spectroscopy (EDS). From the two distinct peaks corresponding to Re and Se can be clearly observed in the EDS spectrum in Fig. 1b, the atomic ratio between Re and Se was 1:2. Stoichiometric analysis of the ReSe_2_ particles was performed via X-ray photoelectron spectroscopy (XPS). The two peaks in the Re 4f_7/2_ and Re 4f_5/2_ spectra (Fig. 2a) at ~ 41 and ~ 43.5 eV, respectively, are consistent with previously reported values^35,36^, while two peaks at ~ 53.9 and ~ 54.8 eV in the Se 3d spectrum (Fig. 2b) signify the presence of Se 3d_5/2_ and Se 3d_3/2_^35,36^, respectively.

**Figure 3:**
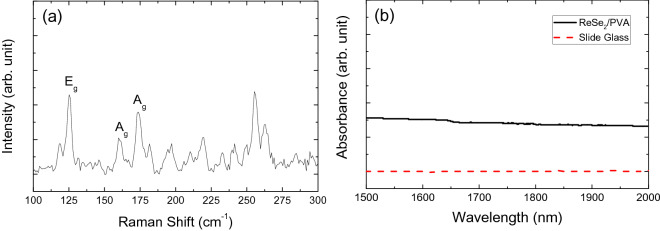
(**a**) A Raman spectrum of the ReSe_2_ particles and (**b**) measured linear absorption spectrum of the ReSe_2_/PVA film.

Raman spectroscopy (Fig. 3a) revealed three peaks corresponding to the E_g_ (~ 125 cm^−1^) and A_g_ (~ 160 cm^−1^ and ~ 173.7 cm^−1^) modes. Many other small peaks in the spectrum can be attributed to the low lattice symmetry and complex lattice vibrations of the particles^35–39^. The size of the ReSe_2_ particles used in this experimental investigation ranged from tens of nanometers to a few micrometers rather than just a few layered evenly sized nanoparticles. To enable the facile formation of a thin film, we combined the ReSe_2_ solution with polyvinyl alcohol (PVA). The ReSe_2_/PVA composite film was then characterized via linear optical absorption (Shimadzu, UV-3600PLUS) measurements after the ReSe_2_/PVA solution had been dropped onto a glass slide and then dried for 1 hour. As shown in Fig. 3b, it is clear that the prepared film had wide spectral band absorption.

**Figure 4:**
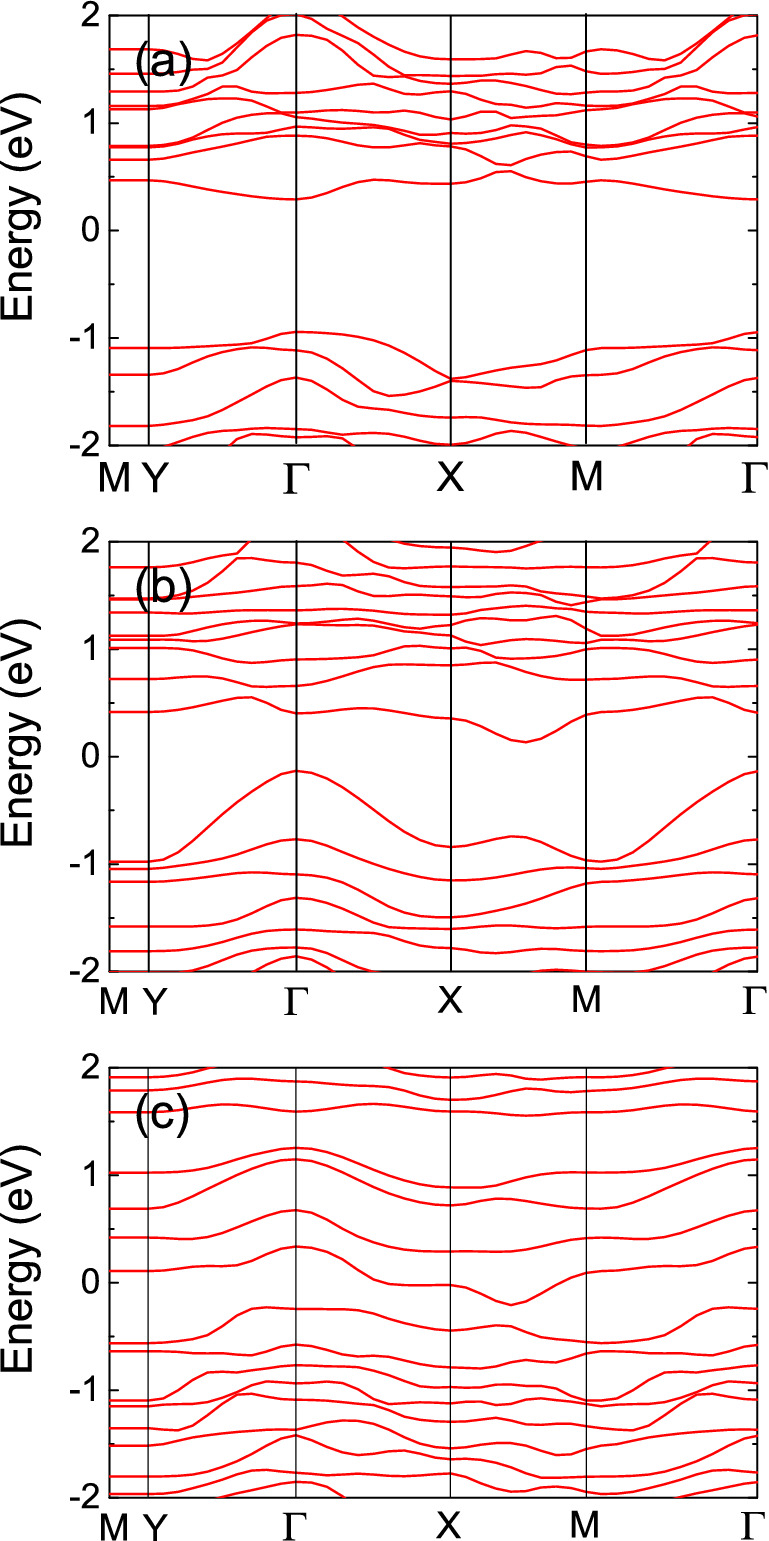
Calculated electronic band structures of ReSe_2_ with (**a**) R = 1:2, (**b**) R = 1:1.75, and (**c**) R = 1:2.667.

To better understand the electronic band structures of ReSe_2_, first-principle calculations were performed using the Quantum Espresso package (Materials square)^40^. We conducted the simulation with different ratios (*R*) of Re and Se atoms (1:2, 1:1.75, and 1:2.667) for which the Perdew–Becke–Erzenhof (PBE) functional was adopted and cutoff-energy of 30 Ry and the ultrasoft pseudopotential were applied. As shown in Fig. 4a, pristine ReSe_2_ with no vacancy has a bandgap of ~ 1.24 eV, which is in good agreement with previously reported values^33,38^. However, the bandgaps of defective ReSe_2_ structures were also calculated with R = 1:1.75 and 1:2.667, as shown in Fig. 4b,c. The indirect bandgap of ~ 0.26 eV, which corresponds to a cut-off wavelength of ~ 4.7 μm, was estimated for R = 1:1.75 whereas a metallic band structure was obtained for R = 1:2.667. These simulation results indicate that the bandgap of ReSe_2_ decreases with an increase in the atomic defect, thereby enabling a broader saturable absorption bandwidth including the mid-infrared wavelength region. A decrease in the defect-induced energy bandgap in TMDC materials has been reported previously^33,41–43^, and the oxidation and defects of TMDCs do not degrade their saturable absorption properties^19,43^. Thus, we can infer that the stability of materials in terms of saturable absorption is not related to oxidation and defects.

### Nonlinear optical property measurements using Z-scan techniques

**Figure 5:**
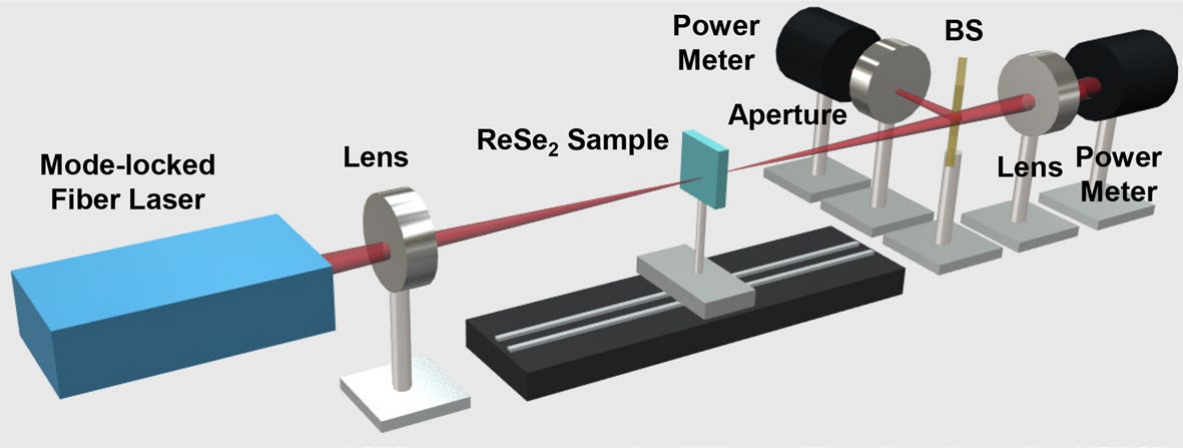
Measured setup for the Z-scan measurements.

**Figure 6:**
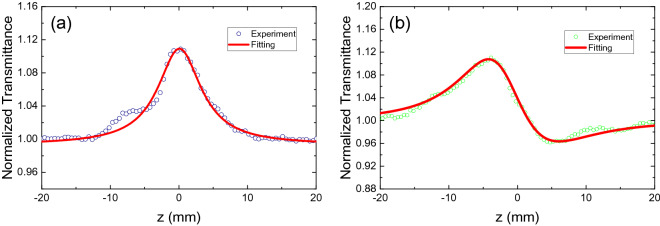
Plots for the (**a**) open-aperture and (**b**) closed aperture Z-scans of the ReSe_2_ film.

The nonlinear optical properties of the ReSe_2_ particles were studied via the Z-scan techniques^44^. Figure 5 shows the Z-scan measurement setup, in which a ~ 550-fs pulsed fiber laser was employed as the input beam. A ReSe_2_/PVA film was vertically placed on the translation stage with a laser output beam focused through the lens onto the film, after which the sample position was moved from z = − 20 to 20 mm. The ReSe_2_/PVA film exhibited an obvious nonlinear saturable absorption response (Fig. 6a); as z approaches 0 (the intensity of the incident beam increased), the normalized transmission was observed to monotonically increase. The transmittance change of the ReSe_2_/PVA film was monitored with a power meter. The normalized transmittance ($T(z)$) of the measured graph was plotted along the z position with a fitted curve based on the following formula^45^:

1 $$ T(z) = \sum\nolimits_{n = 0}^{\infty } {{{( - \beta I_{0} L_{eff} )^{n} } \mathord{\left/ {\vphantom {{( - \beta I_{0} L_{eff} )^{n} } {(1 + {{z^{2} } \mathord{\left/ {\vphantom {{z^{2} } {z_{0}^{2} }}} \right. \kern-\nulldelimiterspace} {z_{0}^{2} }})^{n} }}} \right. \kern-\nulldelimiterspace} {(1 + {{z^{2} } \mathord{\left/ {\vphantom {{z^{2} } {z_{0}^{2} }}} \right. \kern-\nulldelimiterspace} {z_{0}^{2} }})^{n} }}(n + 1)^{{{3 \mathord{\left/ {\vphantom {3 2}} \right. \kern-\nulldelimiterspace} 2}}} } \approx 1 - {{\beta I_{0} L_{eff} } \mathord{\left/ {\vphantom {{\beta I_{0} L_{eff} } {2^{{{3 \mathord{\left/ {\vphantom {3 2}} \right. \kern-\nulldelimiterspace} 2}}} (1 + {{z^{2} } \mathord{\left/ {\vphantom {{z^{2} } {z_{0}^{2} }}} \right. \kern-\nulldelimiterspace} {z_{0}^{2} }})}}} \right. \kern-\nulldelimiterspace} {2^{{{3 \mathord{\left/ {\vphantom {3 2}} \right. \kern-\nulldelimiterspace} 2}}} (1 + {{z^{2} } \mathord{\left/ {\vphantom {{z^{2} } {z_{0}^{2} }}} \right. \kern-\nulldelimiterspace} {z_{0}^{2} }})}}, $$

2 $$ L_{eff} = {{(1 - e^{{ - \alpha_{0} L}} )} \mathord{\left/ {\vphantom {{(1 - e^{{ - \alpha_{0} L}} )} {\alpha_{0} }}} \right. \kern-\nulldelimiterspace} {\alpha_{0} }}, $$

where $\beta$ represents the nonlinear absorption coefficient; $I_{0}$ is the peak intensity at the focusing point; $\alpha_{0}$ denotes the linear absorption coefficient; $z$ and $z_{0}$ denote the position of the film and the Rayleigh length, respectively; $L$ represents the film thickness.

We subsequently conducted a CA Z-scan analysis to measure the nonlinear refractive index of the ReSe_2_/PVA film; Fig. 6b shows the Z-scan results as a fitted curve based on the following formula^46^:

3 $$ T(z) = \frac{1}{{1 - \frac{{4{z \mathord{\left/ {\vphantom {z {z_{0} }}} \right. \kern-\nulldelimiterspace} {z_{0} }}}}{{(1 + {{z^{2} } \mathord{\left/ {\vphantom {{z^{2} } {z_{0}^{2} }}} \right. \kern-\nulldelimiterspace} {z_{0}^{2} }})^{2} }}\Delta \Phi + \frac{4}{{(1 + {{z^{2} } \mathord{\left/ {\vphantom {{z^{2} } {z_{0}^{2} }}} \right. \kern-\nulldelimiterspace} {z_{0}^{2} }})^{3} }}\Delta \Phi^{2} }}, $$

4 $$ \Delta \Phi = {{2\pi n_{2} I_{0} L_{eff} } \mathord{\left/ {\vphantom {{2\pi n_{2} I_{0} L_{eff} } \lambda }} \right. \kern-\nulldelimiterspace} \lambda }, $$

5 $$ z_{0} = {{\pi \omega_{0}^{2} } \mathord{\left/ {\vphantom {{\pi \omega_{0}^{2} } \lambda }} \right. \kern-\nulldelimiterspace} \lambda }, $$

where $\omega_{0}$ is the beam waist, $\lambda$ is the light wavelength, and $n_{2}$ denotes the nonlinear refractive index.

From the measurement results and fitted curves (Fig. 6), the nonlinear absorption coefficient of the ReSe_2_/PVA film was estimated as ~ − 11.3 × 10^3^ cm/GW and its refractive index was ~ − 6.2 × 10^−2^ cm^2^/GW at 1.9 µm. For comparison, the reported nonlinear optical parameters for several other TMDCs are listed in Table 1. Nevertheless, it was not possible to directly compare the measured parameters of ReSe_2_ with the ones of other TMDCs because, to the best of our knowledge, no previous measurements of the nonlinear optical parameters for TMDCs in the 2-μm wavelength area have been reported. The nonlinear absorption coefficient and nonlinear refractive index of ReSe_2_ at 1.9 µm are twice as larger as the values at 1560 nm^34^. In our previous work, we reported that the nonlinear absorption coefficient and nonlinear refractive index of ReSe_2_ are ~ − 5.67 × 10^3^ cm/GW and ~ − 2.81 × 10^−2^ cm^2^/GW at 1560 nm, respectively.

**Table 1:** Comparison of the nonlinear optical parameters of various TMDCs.

TMDC materials	λ (nm)	α_0_ (cm^–1^)	*β* (cm/GW)	n_2_ (cm^2^/GW)	Refs.
Monolayer MoS_2_	532	NA^a^	− 10.11 × 10^5^	NA	^54^
Monolayer MoS_2_	532	NA	− 7.24 × 10^5^	NA	^54^
Monolayer MoS_2_	532	NA	− 1.67 × 10^5^	NA	^54^
Monolayer MoS_2_	532	NA	0.22 × 10^5^	NA	^54^
MoS_2_	800	5.4 × 10^4^	− 136.13	NA	^55^
MoS_2_ (25–27 L)	800	6.24 × 10^4^	11.4 ± 4.3	NA	^56^
MoS_2_ (25–27 L)	1030	3.90 × 10^5^	66 ± 4	NA	^56^
MoS_2_ (72–74 L)	1030	1.89 × 10^4^	− 250 ± 50	NA	^56^
MoS_2_	1064	NA	− 3.8 ± 0.59	(1.88 ± 0.48) × 10^–3^	^57^
WS_2_ (1–3 L)	515	5.18 × 10^6^	(− 2.9 ± 1.0) × 10^4^	NA	^56^
WS_2_	800	8.88 nm^−1^	(− 3.7 ± 0.28) × 10^5^	8.1 ± 0.41	^58^
WS_2_ (1–3 L)	800	1.08 × 10^6^	525 ± 205	NA	^56^
WS_2_ (18–20 L)	800	7.22 × 10^5^	− 397 ± 40	NA	^56^
WS_2_ (1–3 L)	1030	7.17 × 10^5^	(1.0 ± 0.8) × 10^4^	NA	^56^
WS_2_ (18–20 L)	1030	5.98 × 10^5^	(3.28 ± 0.11) × 10^3^	NA	^56^
WS_2_ (39–41 L)	1030	8.57 × 10^5^	(2.75 ± 0.10) × 10^3^	NA	^56^
WS_2_	1064	NA	− 5.1 ± 0.26	(5.83 ± 0.18) × 10^–2^	^57^
WSe_2_	1064	NA	1.9 ± 0.57	− (2.4 ± 1.23)	^57^
Mo_0.5_W_0.5_S_2_	1064	NA	(1.91 ± 0.78) × 10	− (8.73 ± 1.47) × 10^–2^	^57^
MoTe_2_	800	NA	− 7.4 × 10^5^	− 1.24	^59^
SnSe_2_	1030	NA	− 13,596	NA	^60^
ReSe_2_	1560	0.25 × 10^3^	− (5.67 ± 0.35) × 10^3^	− (2.81 ± 0.13) × 10^–2^	^34^
ReSe_2_	1900	1.73 × 10^3^	− 13.8 × 10^3^	− 6.3 × 10^–2^	This work

*NA* not available.

### Fabrication of a ReSe_2_/PVA-based saturable absorber

To determine the practicability of using ReSe_2_ in a device at 1.9 μm, we fabricated a fiberized SA by dropping ReSe_2_/PVA solution onto the surface of a side-polished SM2000 fiber and drying it for 1 day. The physical distance between the flat side and the fiber core was ~ 5 μm, while it had a side-polished section length of ~ 2 mm. After drying, we measured the insertion loss and polarization-dependent loss (PDL) (~ 3.4 dB and ~ 4 dB, respectively). It is possible to reduce the PDL by decreasing the polished section length of a side-polished fiber and/or increasing the distance between the core and the polished fiber surface^47,48^, and thus we tried to use the aforementioned methods to reduce the PDL. However, we observed that the saturable absorption did not proceed very well due to reduced interaction between the deposited ReSe_2_/PVA layer and the oscillating beam.

**Figure 7:**
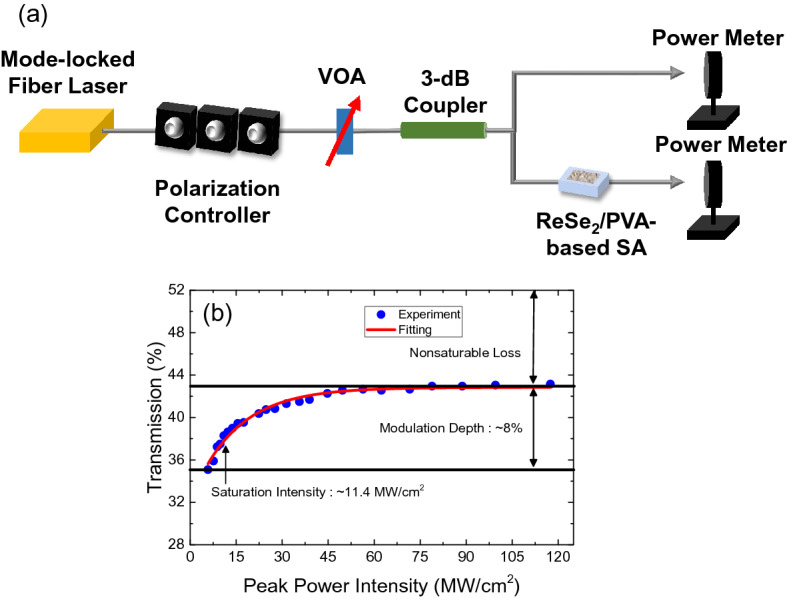
(**a**) Experimental setup for nonlinear transmission measurements and (**b**) the nonlinear transmission curve of the ReSe_2_/PVA-based SA.

Next, we measured the nonlinear transmission of the ReSe_2_/PVA-based SA versus the input beam peak power, for which a 1.9-μm thulium-holmium (Tm–Ho) co-doped fiber laser with a ~ 1.2-ps pulse width was used, as shown in the measurement setup in Fig. 7a. Figure 7b exhibits the measured transmission curve of the ReSe_2_/PVA-based SA, from which the saturation intensity and modulation depth were estimated as ~ 11.4 MW/cm^2^ and ~ 8%, respectively. Hence, the measured modulation depth is high enough for mode-locking in an anomalously dispersive fiber cavity^49,50^.

### Fiber laser setup and results

**Figure 8:**
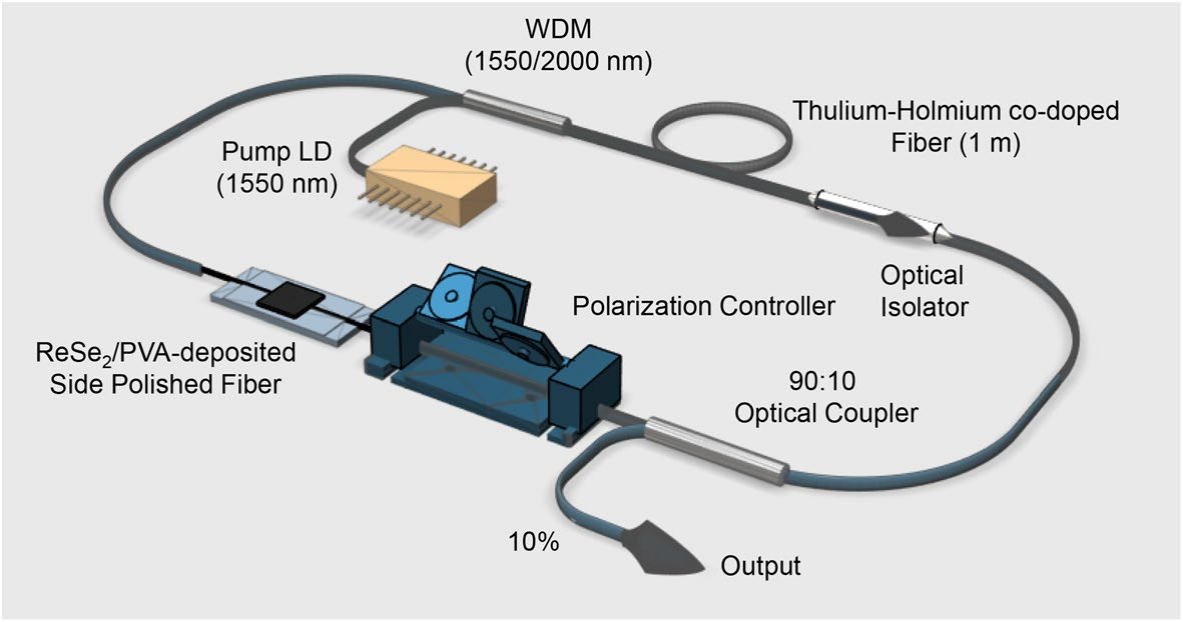
The fiber laser configuration and setup.

The experimental setup of the fiber laser is illustrated in Fig. 8. The gain fiber was 1-m length of co-doped Tm-Ho fiber. Its peak absorption was ~ 13 dB/m at a wavelength of 1550 nm. The pumping source for the cavity was a 1550-nm pump laser diode and the pumping beam was launched into the Tm-Ho co-doped fiber through a 1550/2000 nm wavelength division multiplexer (WDM). A polarization controller (PC) was used to optimize the polarization state of the beam, while an isolator was placed in the cavity for unidirectional light propagation and the SA was inserted between the WDM and the PC. The output pulses were extracted through a 90:10 optical coupler.

Self-started optical pulses were induced at the pump power of ~ 224 mW. Our SA had a relatively high PDL, and so we believe that the mode-locking pulses were generated from the fiber laser cavity through the combined use of saturable absorption and NPR. The self-starting capability of our laser was tested by repeatedly increasing and decreasing the pump power near ~ 224 mW. The self-starting phenomenon was clearly observed, which we believed is due to the relatively slow response of the saturable absorption owing to the presence of ReSe_2_. Anyhow, it is very difficult to negate the contribution of NPR to the laser mode-locking due to the 4 dB PDL value of the SA. Although it is difficult to realize self-starting mode-locking in fiber lasers with NPR mode-locking, it can be readily achieved in saturable absorption mode-locking. In this experiment, the self-starting operation was easily achieved without the necessity of a precise PC adjusting procedure. Therefore, we consider that the mode-locking demonstrated in this experiment is a hybrid type of saturable absorption and NPR.

**Figure 9:**
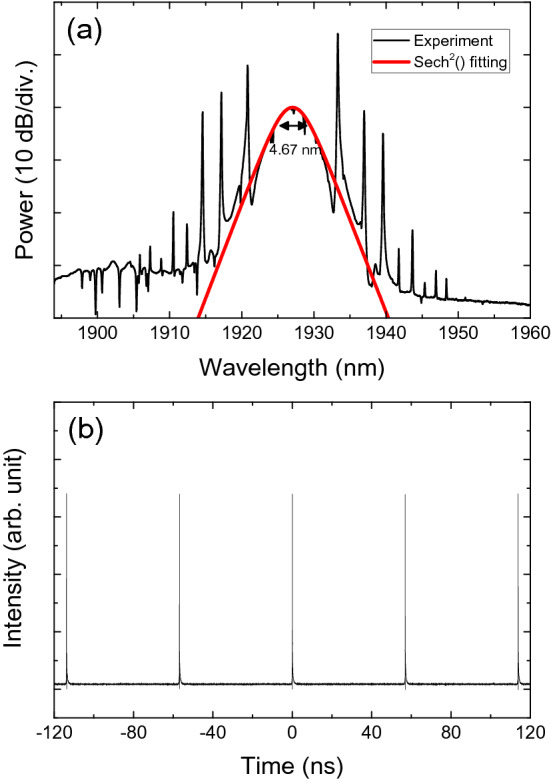
(**a**) An optical spectrum and (**b**) an oscilloscope trace of the output pulses at a pump power of ~ 297 mW.

The measured optical spectrum with its hyperbolic secant function curve is illustrated in Fig. 9a. The 3-dB bandwidth is ~ 4.67 nm with a center wavelength of ~ 1927 nm. Many Kelly sidebands were observed; it is well-known that these result from the periodic spectral interference of soliton pulses and dispersive waves in the laser cavity, depending on the phase-matching conditions. Therefore, it was possible to generate many Kelly sidebands when the phase-matching conditions were well satisfied in the laser cavity. Note that there are several reports on obtaining many Kelly peaks in passively mode-locked Tm-doped fiber lasers^51–53^. The temporal shape and spectrum of the output pulses exhibited little change when the pump power was changed from 224 to 297 mW. The measured period of the pulse train was ~ 56.92 ns (Fig. 9b) that corresponded to a repetition rate of ~ 17.57 MHz, which is a fundamental resonance frequency of the cavity.

**Figure 10:**
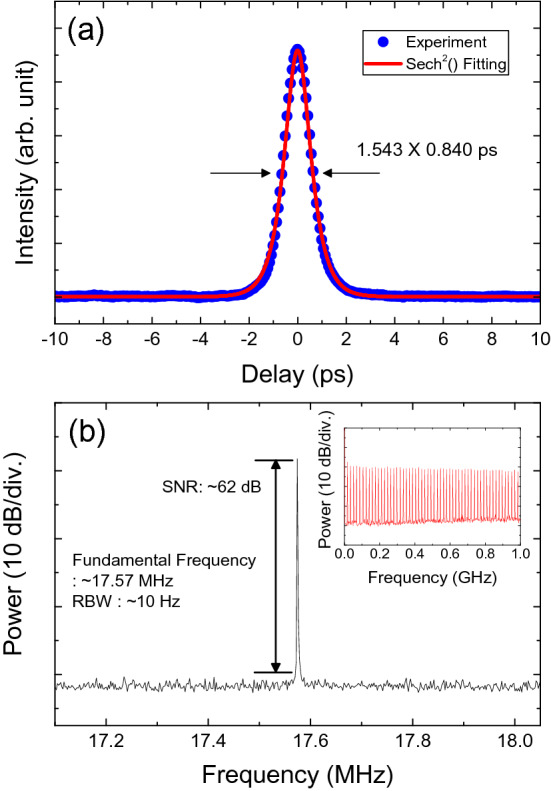
(**a**) An autocorrelation trace and (**b**) an electrical spectrum. Inset: the electrical spectrum over 1 GHz at a pump power of ~ 297 mW.

We subsequently performed autocorrelation measurements of the output pulses (Fig. 10a), for which the output temporal width was measured at ~ 840 fs. Considering the 3-dB bandwidth of ~ 4.67 nm, the time-bandwidth product was calculated as ~ 0.317, which indicates that the pulses are almost transform-limited. Figure 10b depicts the radio frequency (RF) spectrum of the output pulses; the signal-to-noise ratio (SNR) was measured as ~ 62 dB with a fundamental frequency of ~ 17.57 MHz. A 1-GHz-span electrical spectrum was also measured (Fig. 10b), in which the strong harmonic signals imply that the laser output consisted of stable mode-locked pulses at the fundamental resonance frequency.

**Figure 11:**
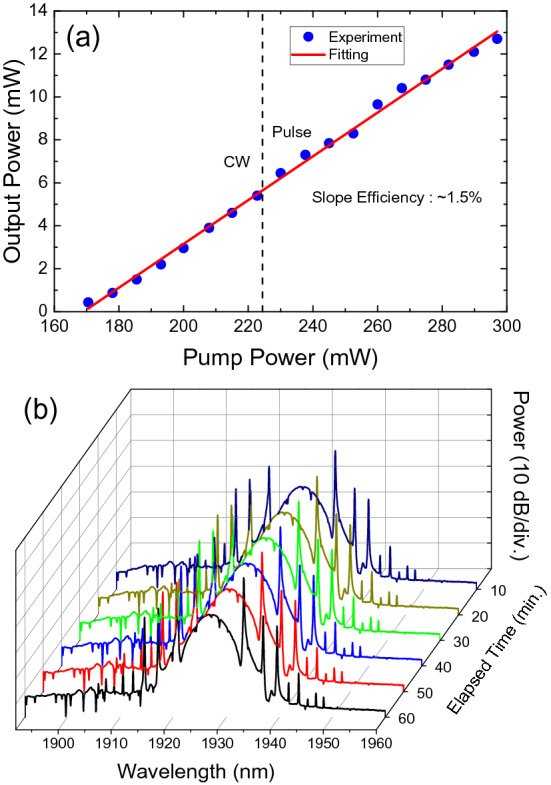
(**a**) Pump output characteristics with continuous wave and pulse regions shown. (**b**) Measured output optical spectrum with a pump power of ~ 297 mW for 1 h.

Figure 11a demonstrates the relationship between the average power of our laser output and the pump power, in which we can see the linear increase in output power with the increase in pump power. The maximum output power was ~ 12.7 mW, and the slope efficiency was ~ 1.5%. Stable output pulses existed at a pump power range from ~ 224 to ~ 297 mW.

Finally, the long-term stability of the pulse laser was examined by measuring the output spectrum every 10 min for 1 h with the pump power set at ~ 297 mW (Fig. 11b). It can be indirectly inferred from the measurements that stable mode-locking was maintained for the duration of the experiment.

## Conclusion

We performed the Z-scan measurements at 1.9 µm to investigate the nonlinear optical responses in ReSe_2_. From the results, the nonlinear absorption coefficient of ReSe_2_ was approximately − 11.3 × 10^3^ cm/GW and the nonlinear refractive index was around − 6.2 × 10^−2^ cm^2^/GW. Furthermore, through energy band structure calculations with the PBE functional, we showed that the bandgap of ReSe_2_ decreases with an increase in atomic defects, allowing for a broader saturable absorption bandwidth that can cover the mid-infrared wavelength region. Moreover, we experimentally demonstrated hybrid mode-locking with a 1.9-μm fiber laser with a ReSe_2_/PVA-based SA. Due to both nonlinear saturable absorption and the high PDL of the prepared SA, 840-fs mode-locked pulses could be generated from a co-doped Tm-Ho fiber laser ring cavity.

We believe that this investigation will be technically meaningful from the viewpoint of providing useful data for producing promising nonlinear optical materials that could be used in the implementation of nonlinear optical and photonic devices. Further investigations need to be conducted to fully understand the nonlinear optical properties of ReSe_2_ in the mid-infrared spectral region beyond 2 μm.

## Methods

### Z-scan measurement

A 1.2-ps fiber laser operating at 1.9 μm was used as the input pulse. The input beam was focused through a plano-convex lens into the ReSe_2_ sample, and the beam passing through the ReSe_2_ sample was separated through a beam splitter. The sample was gradually moved in the propagation direction. One of the two separated beams was used for the OA Z-scan while the other passed through the aperture and was used for CA Z-scan. Power meters were used to measure the varying power of the beam while the sample moved.

### Nonlinear transmission curve measurement

The fiber laser used in the Z-scan measurements was used as the input source that was adjusted using a variable optical attenuator. After a 3-dB coupler, the input pulses were separated into two ports. One was directly detected by a power meter to measure the reference beam power while the other was connected to an SA. Another power meter was connected to the prepared SA to monitor the output power for comparison with the input power. A PC was employed within the setup to show the non-negligible PDL of the SA.
